# Surgical treatment of bronchopulmonary malformations in children: experience in a Brazilian center

**DOI:** 10.1590/0100-6991e-20253801-en

**Published:** 2025-02-13

**Authors:** ÁTILA MAGALHÃES VICTÓRIA, FABIO BOTELHO, CLECIO PICARRO, PAULO CUSTÓDIO FURTADO CRUZEIRO, SHERIF EMIL, JOSÉ CARLOS FRAGA, MARCELO ELLER MIRANDA

**Affiliations:** 1 - Hospital das Clínicas da UFMG/EBSERH, Cirurgia Pediátrica - Belo Horizonte - MG - Brasil; 2 - McGill University, Pediatric Surgery - Montreal - Quebec - Canadá; 3 - Faculdade de Medicina da Universidade Federal de Minas Gerais (UFMG), Departamento de Cirurgia - Belo Horizonte - MG - Brasil; 4 - Montreal Children’s Hospital - McGill University, Pediatric Surgery - Montreal - Quebec - Canadá; 5 - Faculdade de Medicina da UFRGS, Cirurgia Pediátrica - Porto Alegre - RS - Brasil

**Keywords:** Bronchopulmonary Sequestration, Cystic Adenomatoid Malformation of Lung, Congenital, Thoracic Surgery, Child, Postoperative Care, Sequestro Broncopulmonar, Malformação Cística Adenomatoide Congênita Do Pulmão, Cirurgia Torácica, Criança, Cuidados Pós-Operatórios

## Abstract

**Introduction:**

Bronchopulmonary malformations (BPM) are lower respiratory tract anomalies that include congenital malformations of the pulmonary airways (CMPA), bronchogenic cysts (BC), bronchopulmonary sequestrations (BPS), and congenital lobar emphysema (CLE). Prenatal detection in low- and middle-income countries is less common than in high-income ones. This study aims to show the experience in the surgical approach to BPM in a Brazilian center, with emphasis on clinical evolution and surgical results, according to the time of diagnosis (prenatal versus postnatal).

**Methods:**

We retrospectively analyzed medical records of patients under the age of 18 who underwent surgery for BPM at a referral center in a middle-income country between 2000 and 2021. Based on the time of BPM diagnosis, we divided the children into two groups: prenatal and postnatal. These groups were evaluated in terms of age at surgery, history of pneumonia before the operation, surgical outcomes (perioperative and postoperative complications, duration of mechanical ventilation, duration of chest tube, length of hospital stay), and histological type of BPM.

**Results:**

In the cohort of 66 patients, 43 (65.1%) had a prenatal diagnosis of BPM, while 23 (34.8%) were identified after birth. Compared with patients diagnosed prenatally, those diagnosed after birth underwent surgery at a higher age (mean of 978 days ± 1341.0 versus mean of 200 days ± 360.9; p<0.01), and had a higher incidence of pneumonia before surgery (65% vs. 12%, p < 0.01). There was no association between the time of BPM diagnosis and postoperative outcomes. All patients with BPS were in the prenatal group, and all patients with CLE were in the postnatal one. There was a higher prevalence of CMPA in the prenatal group compared with the postnatal one (72% vs. 39%, p < 0.01).

**Conclusion:**

In a Brazilian center, approximately 2/3 of the patients had an intrauterine diagnosis of bronchopulmonary malformations and were treated early at a neonatology center. Patients diagnosed with BPM only after birth were more likely to have pneumonia and undergo surgery at an older age than patients with an intrauterine diagnosis. Prospective, multicenter studies, including asymptomatic patients treated conservatively, without surgical interventions, and patients operated by video-assisted thoracoscopy, would be well indicated to evaluate the future evolution of children with BPM and to establish protocols appropriate to the Brazilian reality.

## RESUMO

**Introdução:**

As malformações broncopulmonares (MBP) são anomalias do trato respiratório inferior que incluem malformações congênitas das vias aéreas pulmonares (MCVAP), cistos broncogênicos (CB), sequestros broncopulmonares (SBP) e enfisema lobar congênito (ELC). A detecção pré-natal em países de baixa e média renda é menos comum do que em países de alta renda. Este estudo pretende mostrar a experiência na abordagem cirúrgica da MBP em um centro brasileiro, com ênfase na evolução clínica e nos resultados cirúrgicos, conforme a época do diagnostico (pré-natal versus pós-natal).

**Métodos:**

Uma análise retrospectiva dos prontuários médicos foi realizada para pacientes com menos de 18 anos que foram submetidos à cirurgia para MBP em um centro de referência de um país de renda média entre 2000 e 2021. Com base no momento do diagnóstico da MBP, as crianças foram divididas em dois grupos: pré-natal e pós-natal. Esses grupos foram avaliados quanto à idade na cirurgia, histórico de pneumonia antes da operação, resultados cirúrgicos (complicações peri- e pós-operatórias, duração da ventilação mecânica, duração do dreno torácico, tempo de internação) e o tipo histológico de MBP.

**Resultados:**

Na coorte de 66 pacientes, 43 (65,1%) tinham diagnóstico pré-natal de MBP, enquanto 23 (34,8%) foram identificados após o nascimento. Em comparação com os pacientes diagnosticados no pré-natal, aqueles diagnosticados após o nascimento foram submetidos à cirurgia com idade maior (média de 978 dias ± 1341,0 versus média de 200 dias ± 360,9; p<0,01), e apresentaram maior incidência de pneumonia antes da cirurgia (65% vs. 12%, p < 0,01). Não houve associação entre o momento do diagnóstico de MBP e os resultados pós-cirúrgicos. Todos os pacientes com SBP estavam no grupo pré-natal, e todos os pacientes com ELC estavam no grupo pós-natal. Houve maior prevalência de MCVAP no grupo pré-natal em comparação com o grupo pós-natal (72% vs. 39%, p < 0,01).

**Conclusão:**

Em um centro brasileiro, cerca de 2/3 dos pacientes tiveram o diagnóstico intrauterino das malformações broncopulmonares e foram atendidos precocemente em centro de neonatologia. Os pacientes com diagnóstico das MBP somente após o nascimento tinham maior probabilidade de apresentar pneumonia e passar por cirurgia com idade maior do que os pacientes com diagnóstico intrauterino. Estudos prospectivos e multicêntricos, incluindo pacientes assintomáticos tratados conservadoramente, sem intervenções cirúrgicas, e os pacientes operados por videotoracoscopia, seriam bem indicados para se avaliar futuramente a evolução das crianças com MBP, e estabelecer protocolos adequados à realidade brasileira.

## INTRODUCTION

Bronchopulmonary malformations (BPM) result from irregular fetal development of the lower respiratory tract^1^ ^,^ ^2^. Based on radiological and histological characteristics^3^, they are categorized into specific types, such as congenital malformation of the pulmonary airways (CMPA), congenital lobar emphysema (CLE), bronchopulmonary sequestration (BPS), and bronchogenic cyst (BC). Some hybrid cases have attributes of both CMPA and BPS^3^. Although previously considered rare, these malformations are now more frequently diagnosed^4^. Recent research estimates that the occurrence of BPM is approximately one in every 2,000 to 2,500 live births, a significant increase from estimates in 2011, of one in 25,000 to 35,000 live births^5^ ^,^ ^6^. This increase is likely due to improvements in obstetric care, antenatal ultrasound (US) technology, and the availability of fetal nuclear magnetic resonance imaging^5^ ^,^ ^7^.

Treatment of these lesions can begin during intrauterine life. In addition to diagnosis, prenatal ultrasound allows measurement of the volume ratio of CMPA. This measure has a robust prognostic value and can guide prenatal interventions, such as maternal corticosteroid administration, thoracoamniotic drainage, fetal lung resection, and intrapartum extrauterine treatment (a technique known as EX utero Intrapartum Treatment - EXIT)^7^.

Children with BPM may remain asymptomatic, experience respiratory failure soon after birth, or develop pneumonia later in life. Treatment varies based on the nature and size of the lesion and associated symptoms^8^. While most of these children are born without symptoms, some may require immediate medical or surgical attention, especially in situations involving respiratory distress or pulmonary infection^8^. Standard surgical therapy for symptomatic children typically involves lobectomy or lung segmentectomy. However, there is an ongoing debate about the approach in asymptomatic children, with some authors indicating resection of lesions, and others, only clinical observation^9^.

Few studies in medical literature on the outcomes of BPMs come from low- and middle-income countries, as classified according to the World Bank: (https://datahelpdesk.worldbank.org/knowledgebase/articles/906519-world-bank-country-and-lending-groups). 

In this context of limited evidence and existing debates, this study aims to examine the outcomes of these surgically treated conditions in a middle-income country, comparing patients who received a prenatal diagnosis with those who received the diagnosis only in the postnatal period.

## METHODS

We included all pediatric patients under 18 years of age who underwent resection of pulmonary lesions pathologically confirmed as bronchopulmonary malformations (BPM) at a referral center of the Public, Unified Health System (SUS) in a middle-income country between January 2000 and December 2021. The center acts as a reference in neonatal care, fetal medicine, and high-risk pregnancy, offering pediatric hospital surgery and fetal magnetic resonance imaging. We excluded patients with incomplete data or who underwent pulmonary resection for other diagnoses. 

Variables collected from medical records included demographic information and surgical outcomes. Specifically, were obtained the demographic and clinical variables place of birth (referral center or other unit), sex, time of diagnosis (prenatal or postnatal), presence of other congenital anomalies, surgical age, location of the lesion, and pathological outcome. The outcomes analyzed included chest tube duration, duration of mechanical ventilation, length of hospital stay, perioperative and postoperative complications, and mortality.

To facilitate the presentation of the results, we described complications according to the Clavien-Dindo classification^10^, which is a system used to categorize surgical complications based on their severity. It is divided into five grades: Grade I involves mild complications that do not require specific treatment; Grade II involves simple pharmacological treatments; Grade III includes complications requiring surgical, endoscopic, or radiological intervention; Grade IV refers to life-threatening complications, requiring intensive care; and Grade V corresponds to death. This classification facilitates the comparison between different studies and the performance of meta-analyses, providing a standardized assessment of surgical outcomes. 

We present qualitative characteristics in frequencies and percentages for descriptive purposes, while quantitative characteristics are summarized using mean or median and standard deviation or interquartile range, as appropriate. Inferential analyses explored associations between the time of diagnosis, pneumonia before surgery, type of BPM, surgical age, and surgical outcomes.

We compared two qualitative characteristics with the Pearson’s chi-square test, and more than two, with the Fisher’s exact test. We used the Mann-Whitney test for quantitative variables due to violations of normality and homoscedasticity, as verified by the Shapiro-Wilk and Levene tests. We applied Winsorization for the treatment of extreme values. The database was created using Microsoft Excel 2016. Statistical analyses were performed using R 3.2.5 and MINITAB 14. The level of significance adopted was 5% (p<0.05).

The institutional Ethics in Research Committee approved this study under number 49439121.8.0000.5149.

## RESULTS

Between January 2000 and December 2021, 96 children with suspected BPM, aged 0 to 17 years, underwent surgical treatment at the Pediatric Surgery referral center. Of these, we excluded 14 due to incomplete data, loss of older medical records, and 16 because they had conditions unrelated to BPM (e.g., bronchiectasis), resulting in 66 patients for analysis.

Table 1 presents comparisons of demographic and clinical characteristics between the prenatal and postnatal diagnosis groups. Patients diagnosed in the prenatal period were significantly more likely to have been born at the referral center (84% vs 26%, p<0.01). 

**Table 1:** Comparisons of demographics and clinical characteristics of patients with intrauterine diagnosis versus postnatal diagnosis.

Characteristics of patients, N = 66	Moment of Diagnosis		p-value
	Prenatal	Postnatal	
	N=43 (65,1%)	N=23 (34,8%)	
Birth at the reference center	36 (84)	6 (26)	<0,01^1^
Prematurity	8 (18,6)	4 (17,4)	0,08^2^
Associated anomalies	6 (14)	3 (13)	1,00^2^
Sex (female)	22 (51)	13 (57)	0,67^1^
Pneumonia prior to surgery, N (%)	5 (12)	15 (65)	<0,01^1^
Surgical age, mean ± standard deviation (median); Days	200± 360,9 (14)	978±1341,0 (223)	<0,01^3^
BPM subtypes, N (%)			<0,0091 (IC 95%: 1,4 a 11,7)
Pulmonary Airway Malformation	31 (72)	9 (39)	
Other Subtypes: BC, CLE, IBPS and EBPS	12 (28)	14 (61)	

BC: Bronchogenic Cyst; CLE: Congenital Lobar Emphysema; BPM: bronchopulmonary malformations, EBPS: Extralobar bronchopulmonary sequestration; IBPS: Intralobar bronchopulmonary sequestration. N = number; % = percentage. 1: Pearson’s chi-square test; 2: Fisher’s exact test; 3: Mann-Whitney test.

Regarding the location of the congenital lesions, we observed that six patients had cysts in more than one lung lobe. Thus, of the 43 newborns with an intrauterine diagnosis, 15 (35%) had the disease in the right lower lobe, 15 (35%) in the left lower lobe, nine (21%) in the left upper lobe, four (9%) in the middle lobe, three (7%) in the mediastinum, and one (2%) in the right upper lobe. On the other hand, of the 23 children with a postnatal diagnosis, seven (30%) had the disease in the right upper lobe, five (22%) in the left upper lobe, four (17%) in the right lower lobe, four (17%) in the mediastinum, three (13%) in the left lower lobe, and two (8%) in the middle lobe.

We observed a substantial difference in the incidence of pneumonia before surgery, with the postnatal group exhibiting a higher prevalence (65% vs 12%, p<0.01). 

The anatomopathological diagnosis varied between groups, CMPA being the most prevalent in the prenatal group (72%) and bronchogenic cysts in the postnatal one (35%). Notably, all six patients with pulmonary sequestration (one extralobar and five intralobar) were in the prenatal group. In contrast, all six patients with congenital lobar emphysema were in the postnatal group, as expected. We compared the subtypes of BPM with intrauterine versus postnatal diagnosis (Table 1), as follows: we grouped the subtypes BC, CLE, Extralobar BPS and Intralobar BPS, in view of the small number of each, and compared them with the subtype CMPA. Thus, of the 43 newborns with an intrauterine diagnosis, 31 (72%) had the CMPA subtype, while 12 (28%) had the other subtypes (BC in six, Intralobar BPS in five, and Extralobar BPS in one). Among the 23 children with a postnatal diagnosis, nine (39%) had the CMPA subtype, and 14 (61%), the other subtypes (BC in eight and CLE in six). A p-value of 0.009 indicated a significant association between the BPM subtype and the time of diagnosis. Children with CMPA were four times more likely (95% CI 1.4-11.7) to have received the diagnosis in prenatal care than children with the other BPM subtypes.

Finally, when compared with patients diagnosed prenatally, those diagnosed postnatally underwent surgery later (mean 978 days ± 1,341.0 (median 223 days) versus 200 days ± 360.9 (median 14 days), p<0.01).

Table 2 presents a comparative analysis of the surgical outcomes between the two groups. The rate of perioperative complications was three times higher in the postnatal diagnosis group (22%) compared with the prenatal one (7%), though the difference did not reach statistical significance. The distribution of perioperative and postoperative complications was comparable between groups. Most complications were classified as Clavien-Dindo grade I or II, indicating that, although there were complications, they were predominantly of lesser severity^10^. Bleeding requiring blood transfusions was the most common perioperative complication (13% in the postnatally diagnosed group vs. 7% in the prenatally diagnosed group). The most frequent postoperative complications included surgical wound infections in six patients (9.0%), all in the group with a prenatal diagnosis.

**Table 2:** Comparisons between the time of diagnosis and surgical outcomes.

Postoperative outcomes	Moment of Diagnosis		p-value
	Prenatal	Postnatal	
	N = 43)	N = 23	
Perioperative complications, N (%)	3 (7)	5 (22)	0,11^1^
Postoperative complications, N (%)	21 (49)	8 (35)	0,31^1^
Chest drainage time, mean ± standard deviation, (median); Days	5 ± 3,1 (3)	4 ± 1,3 (3)	0,71^2^
Duration of mechanical ventilation, mean ± standard deviation (median); Days	3 ± 4,9 (1)	6 ± 18,2 (1)	0,95^2^
Length of hospital stay, mean ± standard deviation (median); Days	17 ± 10,2 (16)	22 ± 24,4 (17)	0,91^2^

1: Fisher’s Exact Test; 2: Mann-Whitney test.

Only one patient in the entire cohort, diagnosed in the prenatal period, died. The patient was a premature, low birth weight newborn with an extensive BPM associated with severe cyanotic congenital heart disease. He underwent an emergency left pneumonectomy and died in the immediate postoperative period due to persistent hemodynamic and respiratory instability. The histopathological results of this patient were consistent with CMPA.

## DISCUSSION

In recent decades, children with BPM have shown increased survival rates and reduced morbidity, due to significant advances in diagnostic imaging, prenatal diagnosis, neonatal intensive care, and improved surgical techniques^3^ ^,^ ^4^ ^,^ ^11^. However, data on the outcomes of these conditions in low- and middle-income countries are scarce. In high-income countries, where there is greater availability of resources, most BPMs are diagnosed in the prenatal period, which contributes to the institution of earlier clinical care. This allows for adequate planning of perinatal care, with better prognosis. 

There are fetal surgery centers abroad where some BPM severe cases are surgically treated during intrauterine life, or at the time of birth, as in the EXIT technique^7^ ^,^ ^17^. In this series, fetal pulmonary cyst puncture was performed in only five patients, which already shows some progress in fetal interventions at this medical center. 

According to Kunisack^7^, a prenatal diagnosis of congenital lobar emphysema is possible. However, in this series, the diagnoses of CLE were established only with imaging and anatomopathological examinations after birth.

We conducted this study to investigate the outcomes of BPM in a medical center in Brazil, a middle-income country, and to specifically evaluate the potential role of antenatal diagnosis in clinical outcomes. It is noteworthy that fetal diagnosis has been a reality in this center throughout the study period. The results indicate that BPM prenatal detection is associated with a higher probability of birth in specialized centers and with the performance of earlier surgical interventions. Notably, some patients in the prenatal group received intrauterine interventions. This finding highlights the benefits of early diagnosis, as it facilitates timely treatment, which can be initiated even before birth, in addition to providing adequate allocation of resources and parental counseling^5^ ^,^ ^12^ ^,^ ^13^.

Although this study suggests a lower incidence of pneumonia among patients with a BPM prenatal diagnosis, it is not possible to definitively determine the significance of this trend. There is no data on the number of patients with BPM who may be living asymptomatically, without a specialized evaluation. If these individuals were included, pneumonia rates could differ from the reported sample.

In Brazil, pneumonia continues to be the leading cause of mortality in children under five years of age, and the presence of post-infectious pleural adhesions can complicate surgeries, potentially increasing surgical risks in both pre- and postoperative phases^14^. Meta-analyses indicate that surgeries performed after pulmonary infections tend to have worse outcomes compared with procedures in asymptomatic children^13^ ^,^ ^15^ ^,^ ^16^. In this study, we observed a trend toward a higher incidence of perioperative complications among patients diagnosed postnatally compared with those diagnosed prenatally, with 22% of postnatal diagnosis cases experiencing complications, mainly perioperative bleeding, compared with 7% in the antenatal diagnosis group.

We found no correlation between preoperative pneumonia or the timing of surgery with increased risk of postoperative complications, prolonged hospital stay, or duration of mechanical ventilation and chest drainage. However, the small size of this sample limits the conclusions drawn from the data.

Resection of the lesions is the established treatment for children with symptomatic BPM, but the best approach for those without symptoms is still discussed among specialists^13^ ^,^ ^15^ ^,^ ^16^. The argument in favor of early intervention is based on preventive benefits, such as avoiding complications, recurrent infections, misdiagnosis of malignancy as a benign anomaly, or the rare development of malignant changes over time^5^ ^,^ ^6^ ^,^ ^17^ ^,^ ^18^. However, the risks associated with surgical and anesthetic procedures, especially in infants, should be carefully considered^13^ ^,^ ^19^ ^-^ ^21^. The literature presents varied perspectives on the likelihood of asymptomatic BPM leading to the development of symptoms in childhood. Some studies suggest a low risk of developing symptoms, approximately 3%, while others indicate a much higher probability, with up to 86% of individuals with BPM eventually developing symptoms^17^ ^,^ ^22^. This disparity provides an excellent opportunity for a shared decision-making process with caregivers. 

It is pertinent to recognize that most of the resections in this study were performed by means of open surgery (94%), since most surgeons were not comfortable with the thoracoscopic approach. The minimally invasive thoracic intervention, video-assisted thoracoscopy, performed in four patients, was only available at this medical center from 2006 onwards, with instruments suitable for children over five years of age. This aspect is crucial in the discussion of the risk-benefit profile of surgical intervention in comparison with observation, especially considering the potential long-term consequences of thoracotomy. Studies such as the one conducted by Safa et al. have highlighted the incidence and severity of musculoskeletal deformities, including scoliosis and chest wall anomalies, after thoracic procedures in infants^23^. The findings underscore the importance of considering the morbidities associated with thoracotomy in the risk-benefit discussion.

The occurrence of surgical site infections exclusively among patients who underwent early surgery after prenatal diagnosis prompts a reassessment of antiseptic protocols for infants. It is necessary to continuously monitor the data to determine whether this trend persists, which may require adjustments in clinical practices to improve patient outcomes. In addition, the surgery team at this center is composed of eleven pediatric surgeons, and the selection of some of them to specialize in pediatric thoracic surgery could improve outcomes^24^.

Finally, the length of hospital stay observed in this study can be attributed to several factors. First, patients who underwent early surgery due to symptomatic presentations required a stabilization period before operation. Asymptomatic patients, on the other hand, were scheduled for surgery only after a CT scan, which required sedation. Given that the hospital performed CT scans under sedation only once a week, this protocol inevitably led to prolonged hospital stays. Other factors, such as complex lesions, which required additional discussions with the pulmonology team, and patients who resided in rural areas or remote towns, may have influenced the length of hospital stay. Although most postoperative complications were mild, they occurred in more than 40% of cases. These complications also contributed to longer hospital stays, regardless of their severity. In summary, these combinations of factors - the need for preoperative stabilization, scheduling restrictions for diagnostic procedures, and the incidence of postoperative complications - resulted in the prolonged length of hospital stay observed in this series.

This study is subject to limitations, mainly due to its retrospective nature and exclusive focus on surgically treated children. The samples of each BPM type are small, and the results lack statistical power. Only the data considered assertive and with quality are presented. Incomplete data were not included in this study. However, this is one of the most extensive series of surgically treated patients with BPM in Brazil. 

Another limitation of this study was the absence, in the medical records, of detailed descriptions of how many pneumonia episodes the children sustained in the preoperative period. Therefore, we did not perform an analysis of single versus repeated episodes of pneumonia and surgical outcomes. 

The purpose of this study is to collaborate with other institutions to validate the findings and determine whether the timing of surgical intervention and specific BPM subtypes would independently influence the risk of postoperative complications. In addition, information on the natural history of asymptomatic BPM cases treated non-surgically would have been valuable. The absence of this data limits the ability to generalize the findings beyond the surgical context and the immediate postoperative outcomes. Future research should include BPMs approached non-surgically, to increase understanding of the progression of this disease and inform clinical and surgical strategies.

About 2/3 of the children (65.1%) in this series had an intrauterine diagnosis of BPM, which shows certain advances, but also points to the need for for greater investments in prenatal and perinatal care, to advance survival and reduce morbidities associated with congenital surgical conditions, such as BPM, in Brazil.

## CONCLUSION

This study shows the clinical characteristics and surgical outcomes of pediatric patients diagnosed with BPM, before and after birth, in a Brazilian center. Most children submitted to BPM surgical treatment had an intrauterine diagnosis, which contributed to planned perinatal care and surgical intervention at an earlier age. The observed association between postnatal diagnosis and a higher likelihood of presenting with pneumonia highlights the challenges of late detection in resource-limited settings, potentially leading to delays in surgical interventions. Future research efforts should focus on prospective, multicenter studies, including asymptomatic patients followed without surgical interventions, and patients operated with minimally invasive techniques, with a view to assessing the evolution of children with BPM, to establish protocols appropriate to the Brazilian reality.
